# Transcranial Direct Current Stimulation of Right Dorsolateral Prefrontal Cortex Does Not Affect Model-Based or Model-Free Reinforcement Learning in Humans

**DOI:** 10.1371/journal.pone.0086850

**Published:** 2014-01-24

**Authors:** Peter Smittenaar, George Prichard, Thomas H. B. FitzGerald, Joern Diedrichsen, Raymond J. Dolan

**Affiliations:** 1 Wellcome Trust Centre for Neuroimaging, Institute of Neurology, University College London, London, United Kingdom; 2 Institute of Cognitive Neuroscience, University College London, London, United Kingdom; Radboud University Nijmegen, Netherlands

## Abstract

There is broad consensus that the prefrontal cortex supports goal-directed, model-based decision-making. Consistent with this, we have recently shown that model-based control can be impaired through transcranial magnetic stimulation of right dorsolateral prefrontal cortex in humans. We hypothesized that an enhancement of model-based control might be achieved by anodal transcranial direct current stimulation of the same region. We tested 22 healthy adult human participants in a within-subject, double-blind design in which participants were given Active or Sham stimulation over two sessions. We show Active stimulation had no effect on model-based control or on model-free (‘habitual’) control compared to Sham stimulation. These null effects are substantiated by a power analysis, which suggests that our study had at least 60% power to detect a true effect, and by a Bayesian model comparison, which favors a model of the data that assumes stimulation had no effect over models that assume stimulation had an effect on behavioral control. Although we cannot entirely exclude more trivial explanations for our null effect, for example related to (faults in) our experimental setup, these data suggest that anodal transcranial direct current stimulation over right dorsolateral prefrontal cortex does not improve model-based control, despite existing evidence that transcranial magnetic stimulation can disrupt such control in the same brain region.

## Introduction

Electrical stimulation of the human brain has received widespread attention over recent years. It has been used to study the function of healthy cortex [1], connectivity between regions [2], as an avenue for treatment in disorders such as depression, Parkinson’s disease and stroke [3]–[6], and to improve normal function such as in skill learning [7], [8].

Here we used transcranial direct current stimulation (tDCS), a technique whereby two electrodes are placed on the skull and a fixed current level is applied [9]. This technique is reported to increase and decrease the excitability of the neural tissue underlying the anodal and cathodal electrode respectively [8], [9]. A number of studies have suggested that high-level cognition can be improved by anodal stimulation of the prefrontal cortex. Specifically, stimulation of the dorsolateral prefrontal cortex (dlPFC) has been shown to decrease risk-taking [10], improve working memory [11], [12] and improve classification learning [13].

We attempted to influence the process of decision-making through anodal stimulation of the right dlPFC. Decision-making is often dissected into a slow, deliberative, goal-directed component and a fast, automatic, habitual component [14]–[16]. In value-based choice, such a distinction is made as model-based versus model-free control [15], [17]. A model-free system learns a cached value for each action based on reward prediction errors and guides behavior based on these alone, trading a minimum of computational effort against a relative lack of flexibility in adjusting to current goals. Model-based control, by contrast, dynamically computes optimal actions by forward planning, a process that is computationally demanding but allows for flexible, outcome-specific behavioral repertoires [15].

We focused on the right dlPFC based on evidence for its role in model-based processes such as the construction and use of associative models [18]–[20] and the coding of hypothetical outcomes [21]. Work on non-human primates also implicates the dlPFC as a site for convergence of reward and contextual information [22]. Furthermore, we recently showed that right, but not left, dlPFC is necessary for model-based control, evidenced by a reduction in model-based control after disruptive theta-burst transcranial magnetic stimulation to the right dlPFC [23]. Here we sought to enhance, rather than disrupt, model-based control through anodal stimulation. We used a task which has been shown to quantify model-based and model-free control [24]–[26] and tested participants undergoing anodal or Sham tDCS stimulation to the right dlPFC in a double-blind, counterbalanced design. We hypothesized that anodal stimulation would improve model-based control without affecting model-free control, an effect driven by an enhancement of a component process of model-based control subserved by the right dlPFC.

## Materials and Methods

We recruited 23 healthy participants to participate in an experiment over 2 sessions. All participants had normal or corrected-to-normal vision and no history of psychiatric or neurological disorders. One participant was excluded from analysis due to failed stimulation after an increase in resistance from drying electrodes, leaving 22 participants (11 female, mean age ± SD: 22.5±5.3 years, all participants were at least 18 years of age at the time of consent) for analysis.

### Ethics Statement

Written informed consent was obtained from all participants prior to the experiment and the UCL Research Ethics Committee approved the study (project number 3450/003).

### Setup of Experiment and Double-blinding Procedure

Participants were tested on 2 occasions between 3 and 8 days apart, going through the same procedure on each day: after obtaining informed consent we determined the electrode locations, explained the task, guided participants through a short practice session, placed the electrodes on the scalp, turned on stimulation, and started the task. The experiment was double-blind, with both experimenter and participant unaware of the stimulation condition (Active or Sham). This was achieved through a system of blinding codes embedded in the stimulation machine (NeuroConn, Germany). First, co-author GP selected 24 pairs of 5-digit codes, each pair containing one code associated with Active and one code associated with Sham stimulation as programmed into the stimulation machine. These were then permuted such that half the pairs had Active stimulation on session 1 and Sham stimulation on session 2, whereas the other half of pairs had the reversed order. GP kept the unblinded version of the codes and handed the permuted set to PS, who acquired the data. Each participant was assigned a pair in order of testing date. When the participant was prepped for stimulation, their session-specific code was entered into the stimulation machine, which then administered the corresponding Active or Sham protocol without any indication as to the stimulation condition. We tested the participant’s awareness of the stimulation condition at the end of the experiment (see below). PS was deblinded after acquisition of all 23 datasets.

### Task

The task design was based on Daw et al. [24] and identical to Wunderlich et al. [25] except for faster trial timings and a larger number of trials. The task was programmed in Cogent 2000 & Graphics (John Romaya, Wellcome Trust Centre for Neuroimaging and Institute of Cognitive Neuroscience development team, UCL) in Matlab (The Mathworks Inc).

**Figure 1:**
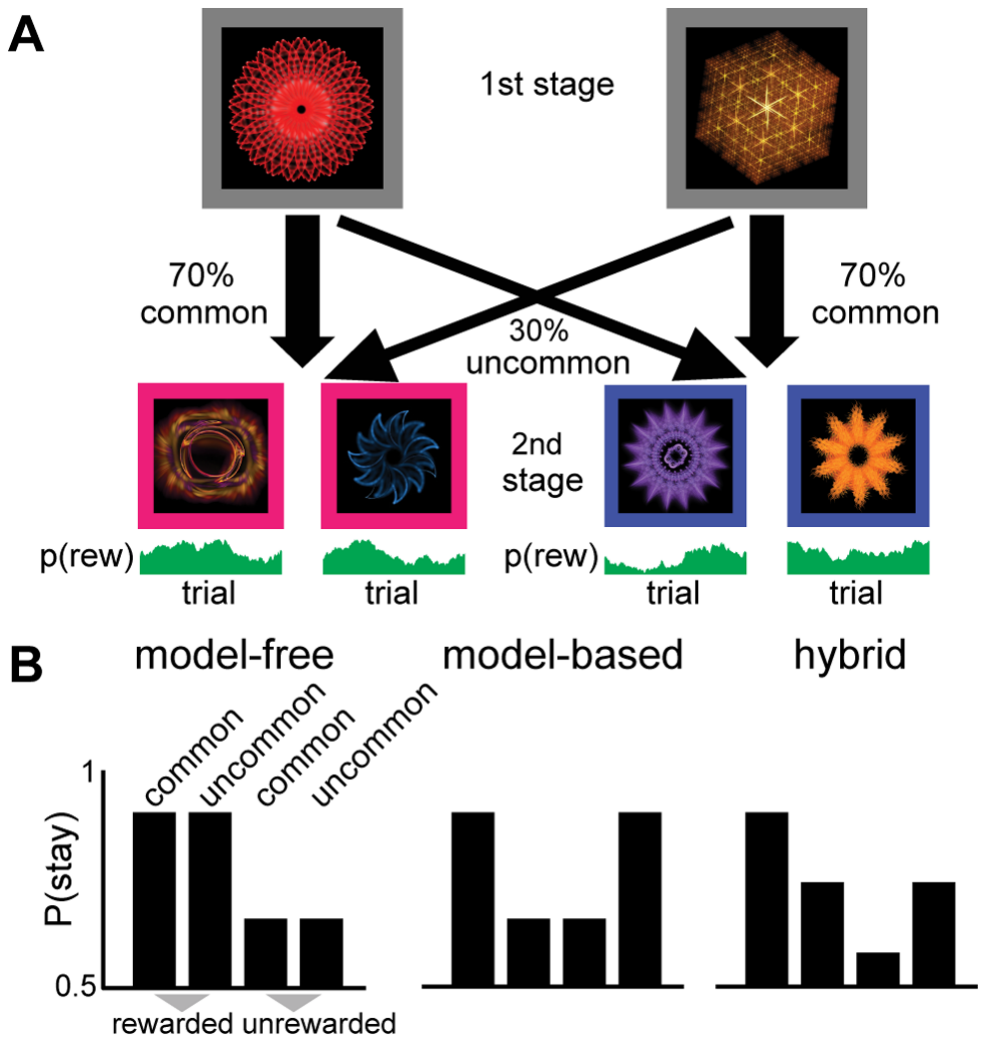
Two-step task design. (A) On each trial a choice between two stimuli led probabilistically to one of two further pairs of stimuli, which then demanded another choice followed probabilistically by reward or no-reward. Participants could learn that each first-stage stimulus led more often to one of the pairs; this task structure could be exploited by a model-based, but not by a model-free controller. (B) Model-based and model-free strategies for reinforcement learning predict differences in feedback processing after uncommon transitions. If choices were exclusively model-free, then a reward would increase the likelihood of staying with the same stimulus on the next trial, regardless of the type of transition (left). Alternatively, if choices were driven by a model-based system, the impact of reward would interact with the transition type (middle).

Each trial consisted of two choice stages. Each choice stage contained a 2-alternative forced choice, with choice options represented by a fractal in a colored box on a black background (Figure 1A). On each choice participants had to respond within 2 seconds using the left/right cursor keys or the trial was aborted and not rewarded. Missed trials were omitted from analysis.

Choice at the first stage always involved the same two stimuli. After participants made their response the rejected stimulus disappeared from the screen and the chosen stimulus moved to the top of the screen. After 0.5 s one of two second stage stimulus pairs appeared, with the transition from first to second stage following fixed transition probabilities. Each first stage option was more strongly (with a 70% transition probability) associated with one of the two second stage pairs, a crucial factor in allowing us to distinguish model-free from model-based behavior (see below). In both stages the two choice options were randomly assigned to the left and right side of the screen, forcing the participants to use a stimulus- rather than action-based learning strategy. After the second choice the chosen option remained on the screen together with a reward symbol (a pound coin) or a ‘no reward’ symbol (a red cross). Each of the four stimuli in stage two had a reward probability between 0.2 and 0.8. These reward probabilities drifted slowly and independently for each of the four second stage options through a diffusion process with Gaussian noise (mean 0, SD 0.025) on each trial. Three random walks were generated beforehand and randomly assigned to sessions. We chose to pre-select random walks as otherwise they might, by chance, turn out to have relatively static optimal strategies (e.g. when a single second-stage stimulus remains at or close to p(reward) = 0.8).

Prior to the experiment participants were explicitly instructed that for each stimulus at the first stage one of the two transition probabilities was higher than the other, and that these transition probabilities remained constant throughout the experiment. Participants were also told that reward probabilities on the second stage would change slowly, randomly and independently over time. On both days, participants practiced 50 trials with different stimuli before starting the task. The main task consisted of 350 trials with 20 s breaks every 70 trials. The participant’s bonus money in pounds sterling was the total number of rewarded trials minus 170, divided by 5. Added to this money was a flat rate of £7/hour.

### Analysis

We analyzed stay-switch behavior on the first choice of each trial to dissociate the relative influence of model-based and model-free control. A model-free reinforcement learning strategy predicts that choices followed by rewards will lead to a repetition of that choice, irrespective of whether it followed a common or uncommon transition (Figure 1B, left). This is because model-free choice works without considering structure in the environment. A reward after an uncommon transition would therefore adversely increase the value of the chosen first stage cue without updating the value of the unchosen cue. In contrast, under a model-based strategy we expect an interaction between transition and reward, because a rare transition inverts the effect of a subsequent outcome (Figure 1B, middle). Under model-based control, receiving a reward after an uncommon transition increases the propensity to choose the previously unchosen first-stage stimulus. This is because the rewarded second stage stimulus can be more reliably accessed by choosing the rejected first stage cue than by choosing the same cue again. To summarize, this analysis quantifies model-free behavior as the strength of the main effect of reward, and model-based behavior as the strength of the reward by transition interaction, even when actual behavior is a hybrid of model-free and model-based control (Figure 1B, right).

Whereas most studies using this task have only looked at the preceding trial to explain choices on the current trial [24]–[26], here we expanded on this approach to examine model-based and model-free influences that go up to 3 trials back. This provides a more fine-grained dissection of the influences of each system on behavior. We used hierarchical logistic regression implemented in lme4 [27] in the R software package. The dependent variable for trial *t* was 1 when stimulus A was chosen and 0 when stimulus B was chosen in the first stage. Each regressor then described whether events on trial t-1, t-2, and t-3 would increase (coded as +1) or decrease (coded as −1) the likelihood of choosing A according to a model-based or model-free system. If a trial contained a common transition the model-based and model-free system would make identical predictions, whereas on trials with uncommon transitions these predictions would be inverted. We additionally modeled the main effect of transition type (common as +1, uncommon as −1) on trial t-1, t-2 and t-3, which we predicted would have no effect on the propensity to choose stimulus A. We also tested 3 alternative models that used 1) one set of model-based regressors for both conditions, 2) one set of model-free regressors for both conditions and 3) one set of model-based and one set of model-free regressors for both conditions (‘null model’). These models allowed us to test whether the additional complexity of having separate regressors for the stimulation conditions was appropriate. These models were compared using the BIC and AIC values provided by the lme4 package.

We estimated coefficients for the regressors shown in Table 1, taking all coefficients as random effects over participants. That is, the regression model is fit to each participant’s data while simultaneously maximizing the likelihood of the parameters across the population. This method accounts for both within- and between-subject variance, providing unbiased estimates of the population coefficient for each regressor. This hierarchical approach is different from the more common approach whereby a full model is fit to each participant separately, and statistics are performed on the parameter estimates. The latter ignores within-subject variance and is only concerned with variance between subjects (i.e. random effects).

**Table 1:** Regressors in the full model for first-stage choices.

regressor	estimate	SE	z-value	p
intercept	0.25	0.03	7.81	**<0.0001**
Active	−264.18	194.46	−1.36	0.1743
Active MF Lag-1	287.02	62.06	4.63	**<0.0001**
Active MF Lag-2	293.64	50.73	5.79	**<0.0001**
Active MF Lag-3	172.87	51.73	3.34	**0.0008**
Active MB Lag-1	244.48	72.35	3.38	**0.0007**
Active MB Lag-2	180.58	66.90	2.70	**0.0069**
Active MB Lag-3	200.76	44.92	4.47	**<0.0001**
Sham MF Lag-1	374.51	51.11	7.33	**<0.0001**
Sham MF Lag-2	287.55	54.85	5.24	**<0.0001**
Sham MF Lag-3	246.79	59.53	4.15	**<0.0001**
Sham MB Lag-1	226.13	64.93	3.48	**0.0005**
Sham MB Lag-2	207.15	77.43	2.68	**0.0075**
Sham MB Lag-3	170.37	60.91	2.80	**0.0052**
Active transition Lag-1	−4.62	36.24	−0.13	0.8985
Active transition Lag-2	9.20	32.34	0.28	0.7760
Active transition Lag-3	−19.03	34.09	−0.56	0.5767
Sham transition Lag-1	−6.61	42.27	−0.16	0.8758
Sham transition Lag-2	15.68	33.42	0.47	0.6389
Sham transition Lag-3	−2.77	36.88	−0.08	0.9400

MF = model-free; MB = model-based; SE = standard error. Lag denotes the effect of time. Bold-face indicates p<.05 uncorrected for multiple comparisons.

We then performed contrasts over the population coefficients to test for differences between conditions in model-free and model-based control. All p-values reported in the manuscript that pertain to the logistic regression were estimated using the “esticon” procedure in the “doBy” package which relies on the chi-square distribution [28]. Power analyses were performed using the Matlab 7.12.0 ‘sampsizepwr’ function and G*Power 3.1.7 [29], [30]. Other tests were performed in SPSS 17.0.

### Stimulation

On both sessions the anodal electrode was placed over right dlPFC and the cathodal electrode over the inion. The inion was chosen for cathodal electrode placement in order to maximize current flow through the dlPFC. The right dlPFC was located using the 10/20 system, which is appropriate given the limited level of spatial resolution of tDCS [31]. In brief, we first located Fpz, Fz and Oz as 10%, 30% and 90% of the nasion-inion distance, measured from the nasion. We then located F8 as 30% of the distance between Fpz and Oz, measured from Fpz passing over the ears. Electrode F4, commonly used for the right dlPFC [31], was then determined as 50% of the distance between F8 and Fz. We used conductive rubber electrodes inserted in a sponge cover measuring 7.5 by 6 cm, secured to the head using a bandage. We placed the electrode along the gyrus, i.e. the electrode was placed in superior-medial to inferior-lateral direction.

We used a DC-stimulator system (NeuroConn, Germany). In the Active condition a 2 mA current was delivered for 25 minutes with 15 s ramping-up and ramping-down. In the Sham condition the current ramped up then down over 15 s, and then performed continuous impedance testing. This manipulation made it very hard for the participant to tell which type of stimulation was given at what time. We confirmed this by giving a 2-alternative forced-choice at the very end of the experiment asking which session contained the Active stimulation. This test showed that participants as a group were not significantly different from chance at determining the session that contained Active stimulation (10 out of 22 participants guessed correctly, binomial test, p = .83). We employed a number of post-hoc checks to safeguard against experimental error. Firstly, we monitored the resistance reported by the DC-stimulator throughout the experiment, rejecting one participant for whom stimulation was stopped after a strong increase in resistance (>55 kΩ). Secondly, after the experiment we confirmed for a random set of 4 sham and 4 active codes that they were correctly linked to the sham or active stimulation procedure by examining the current with an amperometer. This was the case for all 8 codes. Thirdly, we note that of the 100,000 possible codes that can be entered into the DC-stimulator only 200 are allowed, minimizing the possibility of erroneously entered codes.

After turning on stimulation the participant waited for 10 minutes before starting the task in order to ensure the effects of stimulation were fully established [9]. Altogether participants received 25 minutes of stimulation at 2 mA. It is known that cortical excitability changes outlast such stimulation durations by over an hour ([9], though see [32]). The window of stimulation therefore need not fully overlap with the task, and in our design stimulation ended approximately halfway through the task. It should be noted that choices for stimulation parameters are based on studies of motor cortex stimulation. It is possible that these parameters, when used on frontal areas, have different effects. To our knowledge there is no published data on this, though we note our protocol is similar to that of other studies using tDCS on dlPFC [10], [13].

## Results

Participants earned (mean±SD) £8.25±2.56 during Active stimulation and £8.30±2.39 during Sham stimulation (no difference in paired samples t-test, t(21) <1). Participants missed 0.10±0.37% of trials during Active stimulation and 0.09±0.18% of trials during Sham stimulation (no difference in paired sampled t-test, t(21) <1).

**Figure 2:**
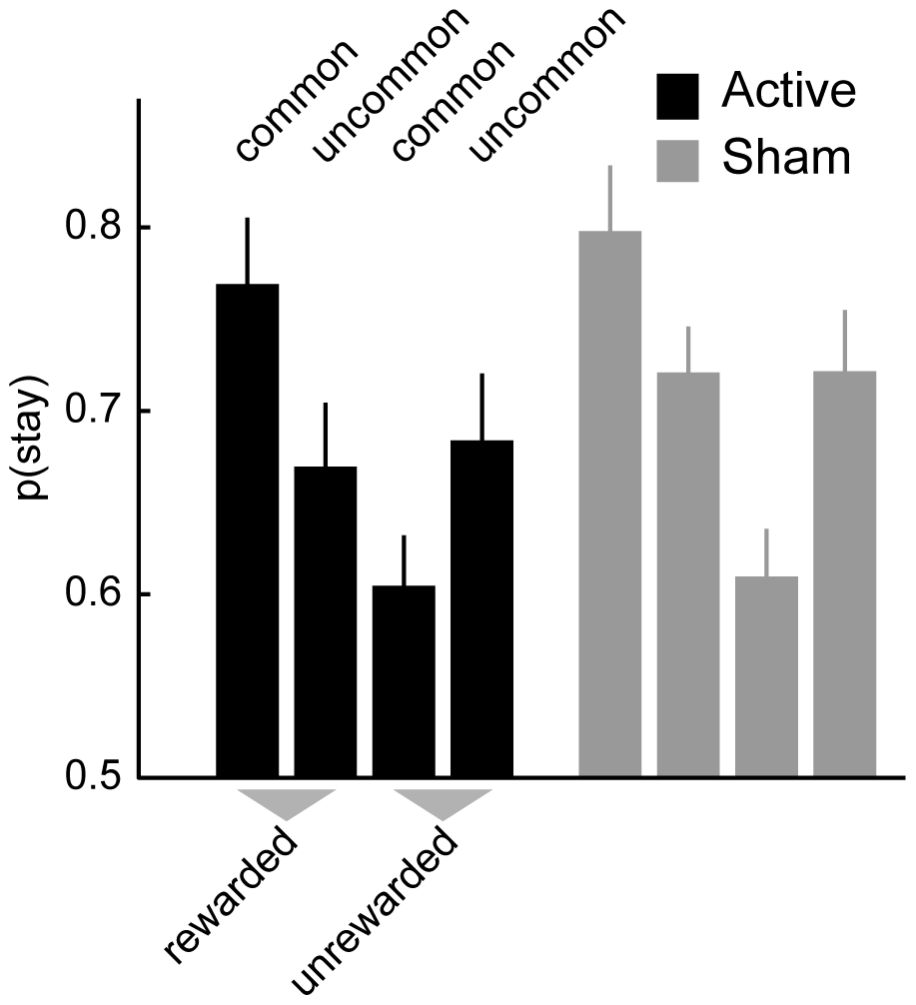
Stay probabilities as a function of reward and transition on previous trial. Participants showed a pattern of stay probabilities characteristic of hybrid model-based/model-free control (cf. Figure 1B) during both Sham and Active stimulation of dlPFC. Error bars indicate SEM.

**Figure 3:**
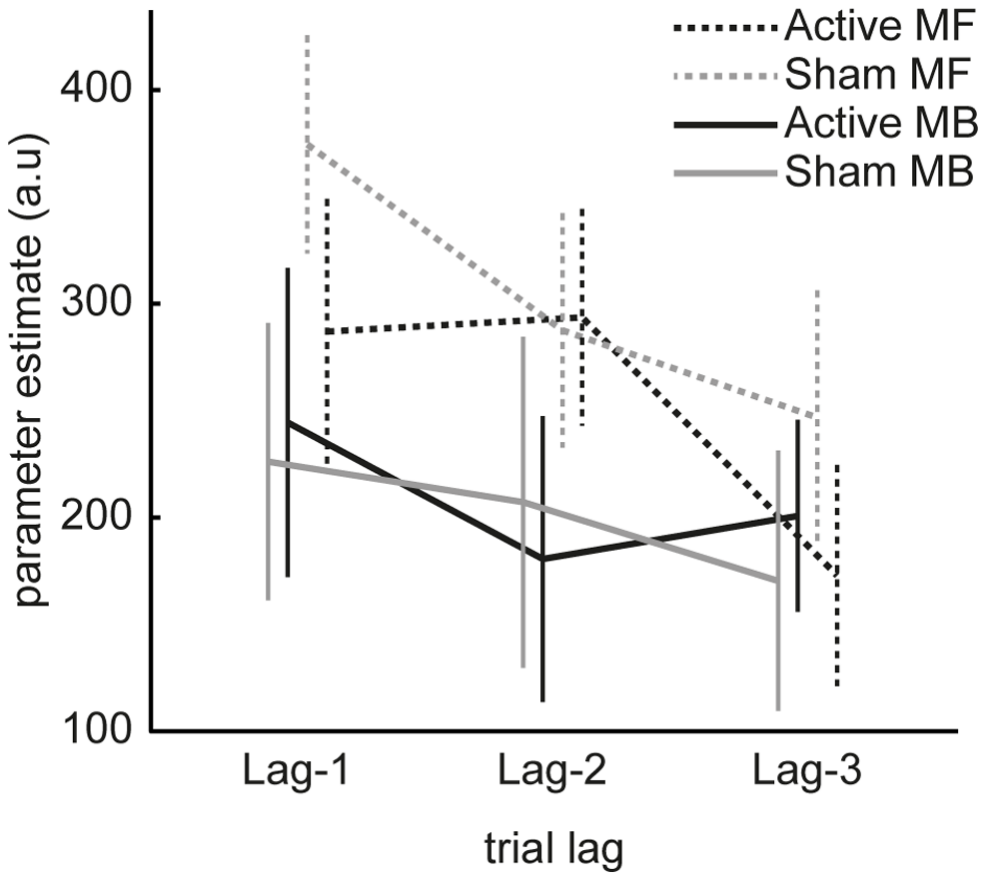
Model-based and model-free influences on choice. We estimated the dependence of a choice at trial *t* on reward and transition events in trials t-1 up to t-3. These regression coefficients can be interpreted as model-based and model-free influences on choice, and larger coefficients indicate a stronger influence over choice. Firstly, all regression coefficients in the plot are significantly larger than zero, suggesting that model-based and model-free systems did not just rely on events on the previous trial but rather on events as far as 3 trials in the past. We did not observe any difference between Active and Sham conditions. Error bars indicate SEM.

For comparison to previous studies using this task we plot the stay probabilities based on reward/no-reward and common/uncommon transition on the previous trial (Figure 2). Qualitatively the pattern in both the Active and Sham condition resembles that of a hybrid controller (Figure 1B, right) in which choices are influenced both by model-based and model-free control. To quantify these influences and examine effects of trials that extend beyond the previous (lag-1) trial, we performed a hierarchical regression analysis (see Table 1 for regressors). This revealed that all model-based and model-free regressors were significantly larger than zero, meaning both systems rely on events at least 3 trials into the past (Figure 3; see Table 1 for statistics). Contrary to our hypothesis we did not find a difference between the Active and Sham stimulation conditions in any of the contrasts (see Table 2 for statistics). We therefore report no evidence for an effect of anodal tDCS to right dlPFC on model-free or model-based control. In subsequent analyses we explored whether this null effect was due to a lack of power in our experiment or due to an inability of tDCS to right dlPFC to modulate model-based or model-free control.

**Table 2:** Contrasts performed on the full model.

Contrast	estimate	SE	?^2^ (1 df)	p
MF Active>Sham	−155.32	119.50	1.69	0.1937
MB Active>Sham	22.17	131.42	0.03	0.8661
MF/MB×Active/Sham	−177.49	192.33	0.85	0.3561
MF Lag-1 Active>Sham	−87.49	55.46	2.49	0.1146
MF Lag-2 Active>Sham	6.09	54.82	0.01	0.9115
MF Lag-3 Active>Sham	−73.93	50.87	2.11	0.1461
MB Lag-1 Active>Sham	18.35	59.86	0.09	0.7592
MB Lag-2 Active>Sham	−26.57	60.15	0.20	0.6587
MB Lag-3 Active>Sham	30.39	54.31	0.31	0.5758
Lag MF Active	114.16	55.61	4.21	**0.0401**
Lag MF Sham	127.72	45.43	7.90	**0.0049**
Lag MB Active	43.72	60.32	0.53	0.4686
Lag MB Sham	55.76	45.04	1.53	0.2157
Lag MF>MB	142.40	124.64	1.31	0.2532
Lag MF Active>Sham	−13.57	65.62	0.04	0.8362
Lag MB Active>Sham	−12.04	70.89	0.03	0.8651
Lag MF/MB×Active/Sham	−1.53	102.76	0.00	0.9882

MF = model-free; MB = model-based; SE = standard error; χ^2^ = chi-square distribution; df = degrees of freedom; Lag denotes the effect of time. Bold-face indicates p<.05 uncorrected for multiple comparisons.

**Figure 4:**
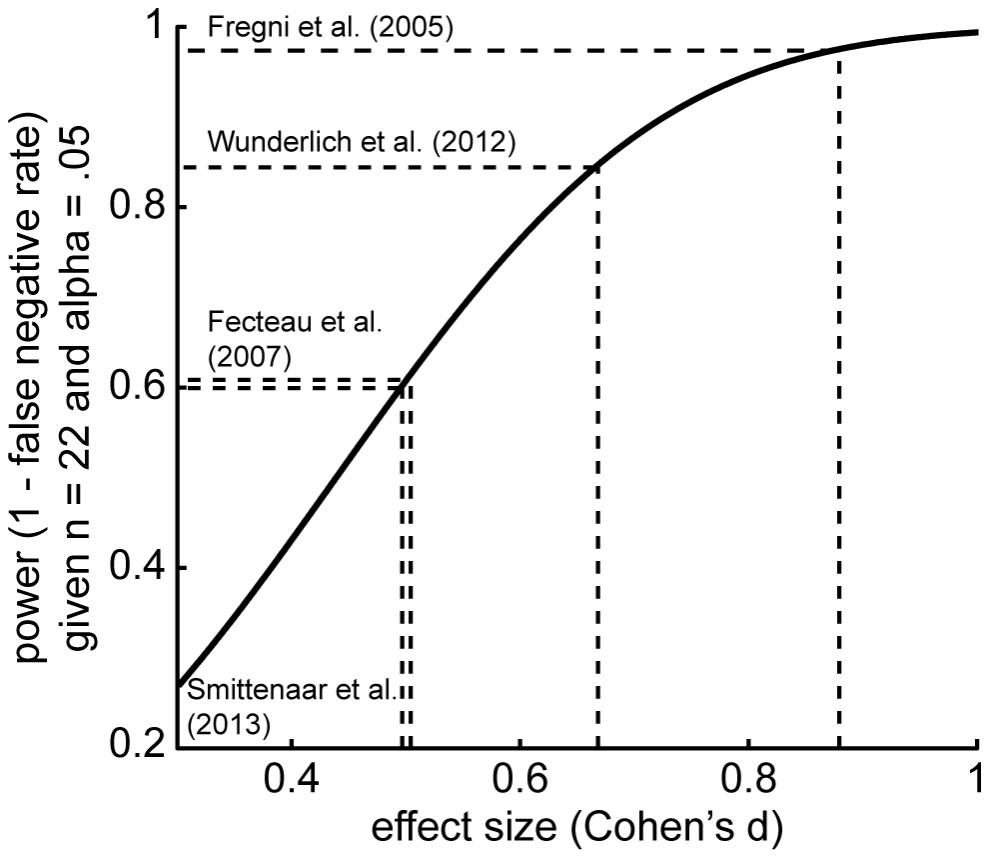
Statistical power to detect true effects. We estimated statistical power in our study based on effect size estimates taken from the published literature. We could then compute the power in our study based on 22 participants and a false positive rate of 0.05 (two-sided alpha). Assuming any true effect of tDCS would have a similar magnitude as the studies shown in the figure, the current study had a power of 50–80%.

To estimate the power in our experiment we gathered effect size estimates in the published literature for manipulations involving the 2-step task [23], [25] and for two tDCS experiments on dlPFC (enhancement of working memory [11]; reduction in risk-taking [10]). We were unable to extract effect size estimates from three other tDCS studies on the dlPFC [13], [33], [34]. For purposes of the power analyses we assumed that a tDCS effect on model-based control has an effect size, expressed in Cohen’s d, similar to these studies. Our power to detect this effect, given a two-tailed alpha of 0.05 and sample size of 22, was then at least 0.60 (Figure 4). Although this is not as high as the normative power of 0.80, it is considerably higher than many studies in cognitive neuroscience [35]. However, to support our claim that tDCS to right dlPFC does not affect model-based and model-free control we formally tested this hypothesis in a model comparison.

The analyses presented above rely on a frequentist approach and hence are framed in terms of null hypothesis testing, which precludes strong conclusions being drawn about the absence of an experimental effect. Hence, based on the preceding analyses we cannot decisively conclude that the null model is more likely compared to the full model that allows for differences in model-free or model-based control in Active versus Sham conditions. Bayesian statistics, by contrast, allow inferences to be made about the absence of experimental effects, and we thus exploited this approach to further probe our results. Thus, we fit three models to the data that were identical to the full model, except that the model-free and/or model-based regressors were assumed identical between stimulation conditions. The first model contained a single set of model-free regressors for both stimulation conditions; the second contained a single set of model-based regressors for both stimulation conditions; and the third (‘null’) contained a single set of model-based and a single set of model-free regressors for both stimulation conditions (see Table 3 for the regressors in the null model). We then performed Bayesian model selection using the Bayesian Information Criterion (BIC) and Akaike Information Criterion (AIC) that are returned by the lme4 package for each model (see Table 4). Although derived within different frameworks, both the BIC and AIC can be thought of as approximations to the true model evidence [36], both containing a term reflecting the likelihood of the model given the data (the ‘accuracy’ term) and a penalization term reflecting the number of parameters in the model (the ‘complexity’ term). As such, the difference in the values of the Information Criteria between models approximates the log Bayes factor, which is the ratio of probabilities of the model given the data. The BIC difference was 900 in favor of the null model when compared to the full model that contains a separate set of model-based and model-free regressors for the Active and Sham condition. This indicates the null model was e^900^ times more likely than the full model. The AIC, which penalizes model complexity less harshly than the BIC, was 100 in favor of the null model compared to the full model, i.e. the null model was e^100^ times more likely. We found a similar pattern of results for the model-free clamped and model-based clamped models which were >e^29^ and >e^44^ less likely than the null model, respectively. Therefore we can conclude that it is significantly more likely that tDCS had no effect on model-based or model-free control than that it did.

**Table 3:** Regressors in the null model which contains the same MB and MF regressors for the Active and Sham stimulation conditions.

regressor	estimate	SE	z-value	p
intercept	0.24	0.03	7.78	**<0.0001**
Active	−269.68	179.42	−1.50	0.1328
MF Lag-1	332.27	48.71	6.82	**<0.0001**
MF Lag-2	285.59	43.58	6.55	**<0.0001**
MF Lag-3	208.50	48.78	4.27	**<0.0001**
MB Lag-1	234.64	61.35	3.82	**0.0001**
MB Lag-2	194.46	64.68	3.01	**0.0026**
MB Lag-3	180.81	45.37	3.99	**0.0001**
Active transition Lag-1	−11.12	35.88	−0.31	0.7566
Active transition Lag-2	7.89	31.01	0.25	0.7993
Active transition Lag-3	−20.15	33.11	−0.61	0.5428
Sham transition Lag-1	0.98	40.73	0.02	0.9809
Sham transition Lag-2	15.32	32.40	0.47	0.6365
Sham transition Lag-3	2.99	35.05	0.09	0.9320

MF = model-free; MB = model-based; SE = standard error. Lag denotes the effect of time. Bold-face indicates p<.05 uncorrected for multiple comparisons.

**Table 4:** Model comparison between a null model (one set of model-based and model-free regressors for both stimulation conditions) and more complex models that allow for an effect of tDCS on model-based control, model-free control, or both, which shows the null model is significantly more plausible than any of the models that allow for an effect of tDCS on behavioral control.

Model	No. of regressorsper subject	BIC	ΔBIC	AIC	ΔAIC	Bayes factor in favor of null model based on AIC
null model	13	18553	0	17752	0	–
separate model-freeregressors for Active and Sham	16	18962	409	17796	44	1.3×10^19^
separate model-basedregressors for Active and Sham	16	18947	394	17781	29	3.9×10^12^
full model	19	19453	900	17852	100	2.7×10^43^

The second column refers to the number of regressors in the hierarchical regression at the individual subject level (cf. Table 1 and 3).

BIC: Bayesian Information Criterion; AIC: Akaike’s Information Criterion.

To test for session effects we performed a hierarchical logistic regression with identical regressors as those described in Table 1, but instead of Active and Sham we coded the regressors as session 1 and 2, respectively. The equivalent contrasts to Table 2 were all p>.15 except effect for Lag on MF in session 1, p = .003, and session 2, p = .06. This suggests that model-based and model-free control do not change with additional exposure to the task, which replicates previous studies [23], [25].

Both model-based and model-free control make equivalent predictions for second-stage choices as there is no task structure to exploit. We nevertheless explored the effects of stimulation on 1-step reward learning. We examined second-stage choices using hierarchical logistic regression similar to our analysis of first-stage choices: stay-switch behavior was regressed against reward received on the most recent trial involving that second-stage pair (i.e. lag-1 only). Transition was not included as a factor because second-stage choices are assumed to be independent of the transition type that led to the state. We observed that in both stimulation conditions there was a main effect of reward, such that if a particular stimulus was rewarded in the most recent encounter with that second-stage pair it was more likely to be chosen again (Active, mean ± SE = 0.96±0.13, p = 9.4×10^−13^; Sham, mean ± SE = 0.82±0.11, p = 5.46×10^−13^). There was a trend-level effect of stimulation-by-reward suggesting a stronger influence of reward under Active stimulation (mean ± SE difference = 0.14±0.08; p = .07), but given the large amount of statistical tests performed we do not further consider this marginal effect. Together, these results suggest stimulation had no effect on second-stage choices.

## Discussion

Here we provide evidence that tDCS to right dlPFC does not affect model-based or model-free control in an established behavioral paradigm. In a double-blind design we confirmed that participants used both model-free and model-based strategies to solve the task, and we could quantify the extent to which either strategy was used. A putative enhancement of right dlPFC activity through Active compared to Sham anodal tDCS stimulation did not significantly change the level of model-based or model-free control. Formally testing this null effect, we provide evidence that a null model predicting no effect of stimulation performed significantly better than more complex models predicting an effect of stimulation on model-based control, model-free control, or both.

We hypothesized that an enhancement of right dlPFC would improve model-based control, similar to beneficial tDCS effects observed on risk taking [10], probabilistic learning [13] and working memory [11]. Based on published tDCS studies and studies of model-based control, we estimated our study had more than 60% statistical power to detect such an effect were it to exist. Although our power was potentially lower than the often cited 80% power standard (e.g. [37]), it was considerably higher than >75% of neuroscience studies as determined recently in a meta-analysis [35]. Despite this, we observed a null effect of tDCS on model-based control. However, frequentist statistics do not allow us to conclude the null hypothesis was a significantly better explanation than the alternatives in which stimulation does have an effect. We therefore performed a complementary model comparison using information-theoretic measures to formally show this [38]. Together, these analyses support our conclusion that tDCS to right dlPFC has no effect on model-based or model-free control.

There is a modest literature on improvement in cognition through tDCS of the right dlPFC, and this begs the question why no effect was found in our experiment. This is even more surprising because the dlPFC is implicated in model-based processes [18]–[22] and when the region is transiently disrupted using transcranial magnetic stimulation, model-based control is selectively impaired [23]. Here we speculate that our null result is most likely due to an inability of tDCS to improve the specific component processes of model-based control subserved by the dlPFC.

Firstly, little is known about the physiological effects of tDCS in prefrontal cortex [39], though this is a rapidly developing field [32]. While there is evidence that anodal stimulation over M1 increases the motor evoked potential (MEP) size elicited by TMS [40], it is not clear how the cellular physiology of the dlPFC is changed following anodal stimulation, nor what the physiological underpinnings of model-based control in the dlPFC are. Despite these unknowns, we suggest here that the neural mechanisms for model-based control in right dlPFC are not amenable to improvement through anodal tDCS.

Secondly, we used a task to assess model-based control that has previously been shown to be susceptible to manipulation [23], [25], [26], we used a set of stimulation parameters that are widely used in the tDCS community [41], and we replicated previous observations of dual control by model-based and model-free systems. Together, this suggests our null result is not due to the introduction of uncertain elements (e.g. novel task or novel stimulation parameters) into the study design.

Despite the use of established methods, we cannot exclude methodological issues as the cause of the null effect altogether. Although we are confident the null effect is not due to faulty equipment or errors in the double-blinding procedure (see Methods), potential other issues might include inaccurate electrode placement, a problem that can be alleviated by stereotactic navigation using anatomical scans as commonly used in transcranial magnetic stimulation [42], and unpredictable current flow based on electrode placement, which might be alleviated by computational models of current flow [43].

We were particularly careful to employ a double-blinded design to eliminate any stimulation-dependent influence from the experimenter on task performance. The task used here requires relatively extensive involvement of the experimenter in the task instructions. In a double-blinded design, then, these effects can be most reliably attributed to the experimental manipulation of interest rather than to unintended information biases [44]. We note that no published work has manipulated the instruction of the 2-step task to examine its influence on model-based and model-free performance.

In conclusion, we provide evidence that anodal stimulation of the right dlPFC by tDCS does not alter model-based or model-free control in our paradigm. This observation was made in the context of extensive and causal evidence for a role of right dlPFC in model-based control in humans. As such, our results should not be interpreted as providing evidence that the right dlPFC is *not* involved in model-based control; rather, our main finding is that anodal stimulation does not necessarily enhance this function. An open question is whether tDCS might improve performance on tasks that are more taxing on the model-based system (e.g. [45]).
